# Nanophotonic devices based on magneto-optical materials: recent developments and applications

**DOI:** 10.1515/nanoph-2021-0719

**Published:** 2022-02-08

**Authors:** Jun Qin, Shuang Xia, Weihao Yang, Hanbing Wang, Wei Yan, Yucong Yang, Zixuan Wei, Wenen Liu, Yi Luo, Longjiang Deng, Lei Bi

**Affiliations:** National Engineering Center of Electromagnetic Radiation Control Materials, School of Electronic Science and Engineering, University of Electronic Science and Technology of China, Chengdu, 610054, China; Microsystem & Terahertz Research Center, China Academy of Engineering Physics (CAEP), Chengdu, 610200, China; Institute of Electronic Engineering, China Academy of Engineering Physics (CAEP), Mianyang, 621900, China

**Keywords:** all-dielectric resonator, biosensor, chiromagnetic metasurface, magnetic field sensing, magneto-optical effects, magnetoplasmonics

## Abstract

Interaction between light and magnetism in magneto-optical (MO) nanophotonic devices has been actively studied in the past few years. The recent development of MO all-dielectric resonators and metasurfaces has led to the emergence of various novel MO phenomena that were not observed in their bulk counterparts. For example, a large s-polarized transverse MO Kerr effect can be observed at magnetic resonance wavelength, which cannot exist in the bare MO films. We review recent developments in nanophotonic devices based on MO materials and focus on different modes and related MO effects in nanophotonic structures with emphasis on recently discovered new MO phenomena in magnetoplasmonics and all-dielectric nanostructures, such as dark mode, all-dielectric Mie resonance and waveguide mode. Further, we discuss the potential applications of these nanostructures for biological/chemical sensing, magnetic field sensing, and magnetic field-controlled active and nonreciprocal metasurfaces.

## Abbreviations

PMOKE: polar magneto-optical Kerr effect
TMOKE: transverse magneto-optical Kerr effect
LMOKE: longitudinal magneto-optical Kerr effect
BIG: bismuth iron garnet
SPR: surface plasmon resonance
LSPR: localized surface plasmon resonance
PSPR: propagating surface plasmon resonance
SLR: surface lattice resonance
LMPIE: longitudinal magneto-photonic intensity effect
FOM: figure of merit
TMPIE: transverse magnetophotonic intensity effect
EIT: electromagnetically induced transparency
BIC: bound state in continuum
LOD: limit of detection
MOSPR: Magneto-optical surface plasmon resonance

## 1 Introduction

Faraday first studied the interaction between light and magnetism in 1845 [1] and Kerr studied it in magnetized materials in 1877, 1878 [2, 3]. They discovered polarization rotation for transmitted (Faraday effect) or reflected light (magneto-optical Kerr effect (MOKE)) when linearly polarized light was incident onto a magnetized medium, referred to as magneto-optical (MO) effects. For the Faraday effect, the polarization rotation angle was proportional to the optical path through the magnetized material. The rotation angle of light propagating along the magnetization direction is dependent only on the direction of the applied magnetic field [4, 5] and is independent of the light propagation direction because of its nonreciprocal nature. Thus, the Faraday effect has been widely utilized in nonreciprocal photonic devices such as optical isolators and circulators [6], [7], [8], [9], [10]. Most magnetic materials exhibit weak MO effects in the visible to the near-infrared frequency range [11], [12], [13], [14]. With the development of integrated and silicon photonics, research is dedicated to the integration of MO materials on waveguide nonreciprocal photonic devices [8, 15], [16], [17]. Further, MOKE found applications in optical data storage, which result in the development of MO discs in the 1980s. In addition, this effect is used for the development of waveguide nonreciprocal photonic devices that leads to phase and intensity nonreciprocity of linear polarized light in MO waveguides, i.e., nonreciprocal phase shift and nonreciprocal loss effects [10, 18], [19], [20], [21].

Given the development of nanophotonic devices, combining magnetic materials with subwavelength photonic nanostructures have become a promising field for enhancing MO effects, discovering novel MO effects, developing active metamaterials and metasurfaces, and enabling the efficient control of magnetism with light [22], [23], [24]. The MO effects and optomagnetism in plasmonic nanostructures comprising MO materials are studied in the field of magnetoplasmonics. A large enhancement of MO effects by the strong near-field localization of the surface plasmon resonance (SPR) modes has been observed [23], [24], [25]. Magnetoplasmonic nanostructures based on pure ferromagnetic metals [26, 27], hybrid noble/ferromagnetic metals [28, 29] and hybrid noble metals/ferromagnetic dielectrics [30, 31] have been studied actively. A strongly enhanced Faraday effect [31], [32], [33], [34], [35], TMOKE [36], [37], [38], [39], [40], and LMOKE [41], [42], [43], [44], [45] have been demonstrated. New MO effects such as the longitudinal magneto-photonic intensity effect (LMPIE) have been experimentally observed [46, 47], and energy-efficient optomagnetic effects have been reported [48], [49], [50], [51], [52]. Further, magnetoplasmonic devices have been proposed for biosensing [53], magnetic field sensing [47], chiral molecule recognition [54], and magnetically tunable nonreciprocal metasurface [55] applications.

A major drawback of magnetoplasmonic devices, which is like other plasmonic devices, is the strong absorption of metals at optical frequencies; this limits the enhancement of the MO effect and results in undesired absorption loss. The recent emergence of optically resonant all-dielectric nanostructures fabricated using low-loss dielectric materials such as Si [56], TiO_2_ [57], and SiN [58] has led to the emergence of all dielectric MO metasurfaces. The first proposed all-dielectric MO nanophotonic device has a subwavelength all-dielectric grating comprising a dielectric MO material. Strong MO effects with high transmittance/reflectance are observed when high-quality factor waveguide modes are excited, and this has been demonstrated experimentally in different material systems [59], [60], [61], [62]. In addition, the violation of Kirchhoff’s law of thermal radiation has been theoretically proposed in grating-coupled MO waveguides [63, 64]. The research interest in discovering new MO effects is triggered by the unique Mie resonance modes in all dielectric resonators. The enhancement of MO effects and the MO figure of merit (FOM) has been demonstrated in hybrid or all-MO dielectric nanoresonators [65, 66]. Nonconventional MO effects such as s-polarized TMOKE have been recently observed [62, 66]. Meanwhile, the MO effects can be significantly enhanced owing to the high-Q resonance in all-dielectric resonators, which leads to the proposal of magnetically controlled active and nonreciprocal metasurfaces [55].

We summarize recent research on nanophotonic devices based on MO materials. We focus on different modes in MO nanophotonic devices with an emphasis on the recent development of novel magneto-nanophotonic structures and the observation of new MO effects in both magnetoplasmonic and all-dielectric MO nanostructures. We discuss the recent development of magnetoplasmonic nanostructures, which features the observation of dark mode-induced strong MO effects, asymmetric/aperiodical plasmonic structures induced multiband MO effect enhancement, observation of optical gyromagnetism, and chiral magnetoplasmonic devices. Next, we move to the MO effects in dielectric nanophotonic devices, and they show that grating-coupled waveguide modes induced giant MO effects, infrared MO nanogratings violating Kirchhoff’s thermal radiation law, and Mie resonance modes induced nonconventional MO effects. Finally, we discuss the potential applications of these MO nanophotonic devices in chemical/biological sensing, magnetic field sensing, and magnetic field-controlled active and nonreciprocal metasurfaces.

## 2 Summary of magneto-optical materials in this review

**Figure 1:**
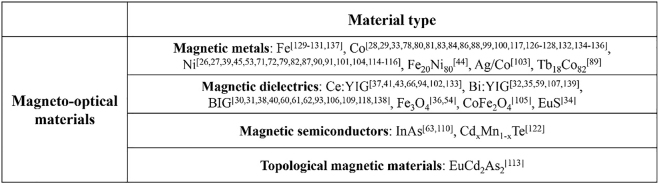
The MO materials reported in this review.

The MO materials appear in this review have been summarized in Figure 1. In general, the magnetic materials used in magneto-nanophotonic devices can be categorized into four groups, including magnetic metals, magnetic dielectrics, magnetic semiconductors and topological magnetic materials. Magnetic metals, such as Fe, Co, and Ni, are usually studied in magnetoplasmonic structures. They show strong MO effects, but also suffer from high absorption loss due to the metallic nature. Magnetic dielectrics, such as Ce:YIG, Bi:YIG, and EuS are studied in magnetoplasmonics and all dielectric resonators. They show high MO effects in the ultraviolet to infrared wavelength range, together with low absorption loss in the near infrared. Magnetic semiconductors, such as InAs, are studied in all dielectric nanostructures. They show strong MO effect in the near to far infrared wavelength range. Recently, topological magnetic materials, such as EuCd_2_As_2_, have attracted great research interest in MO nanostructures. They show particularly strong MO effect in the near infrared to terahertz wavelength range, making them promising candidates for nonreciprocal photonic device applications.

## 3 Magnetoplasmonics

The enhancement of the MO effect and the change in plasmonic resonance modes under applied magnetic fields in plasmonic nanostructures are studied under magnetoplasmonics. Although such effects can be observed in nonmagnetic nanophotonic structures such as pure Au [67], [68], [69], [70] provided the applied magnetic field is sufficiently strong, most magnetoplasmonic devices study plasmonic resonances in hybrid material systems with magnetic and nonmagnetic materials. Another interesting phenomenon of magnetoplasmonics is the phase tuning by MO response, which are both demonstrated in nonperiodic and periodic magnetoplasmonic nanoantennas [71, 72]. In the past decade, extensive research on magnetoplasmonic devices has been reported in several excellent review papers [22], [23], [24], [25, 73], [74], [75], [76], [77]. Here, we briefly review only the different magnetoplasmonic structures from the optical mode point of view, and we focus on several recently developed aperiodical/asymmetric magnetoplasmonic devices that exhibit unique resonance modes and MO effects. For a more systematic review of magnetoplasmonic devices, we refer the reader to references [22, 23, 25, 74, 77].

**Figure 2:**
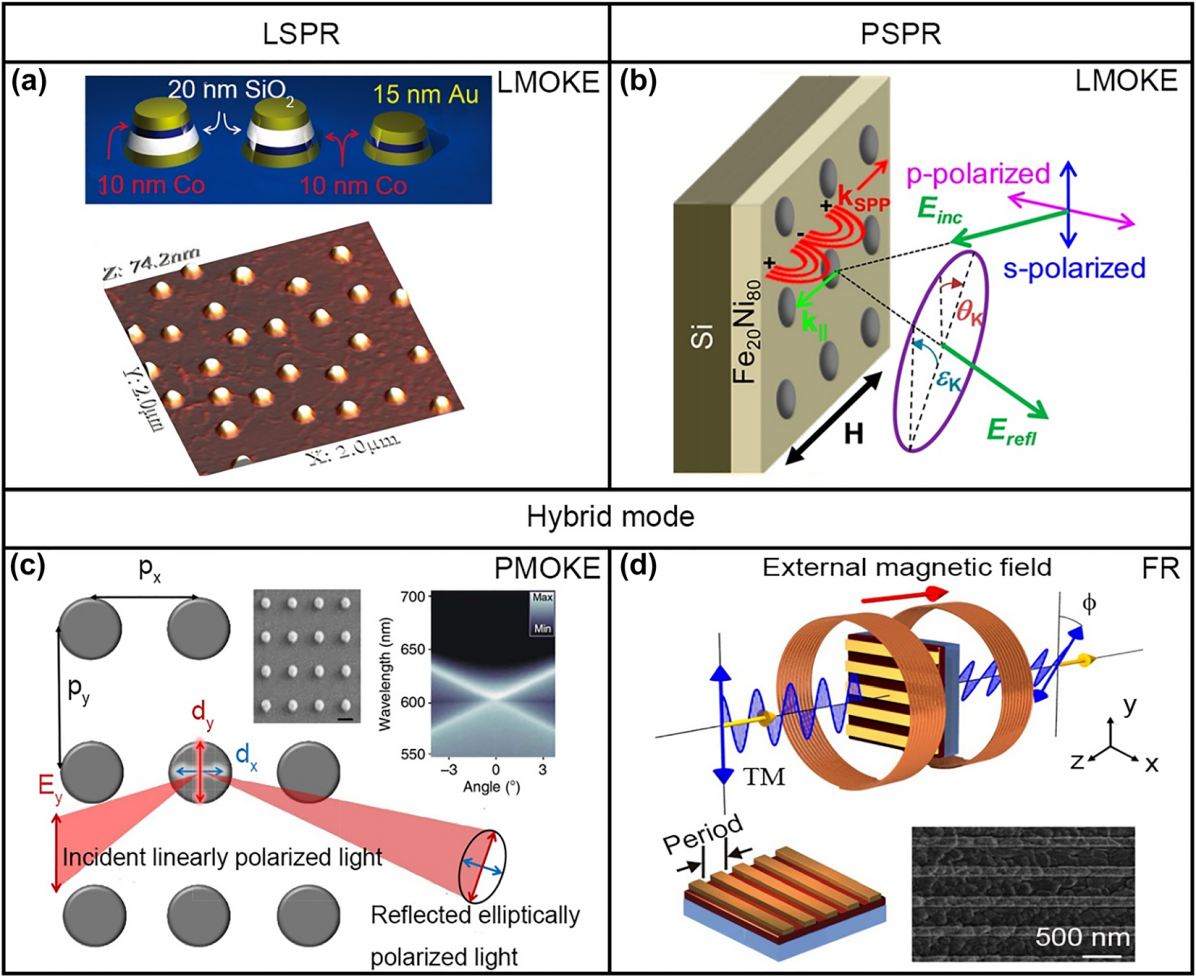
Resonance modes reported in magnetoplasmonics devices. (a) LSPR modes: high MO activity and low optical losses in Au/Co/Au-SiO_2_ sandwich magnetoplasmonic nanodisks. (b) Propagating SPP modes: magnetoplasmonic crystals consist of Fe_20_Ni_80_ periodic nanoholes. (c) Hybrid modes: Ni periodic nanodisk arrays supporting Fano-type resonances induced by hybrid lattice mode and LSPR modes. (d) Hybrid modes: giant enhancement of the MO FR in Au/BIG hybrid magnetoplasmonic structures. Panels of this figure contain pictures adapted from References [28, 31, 44, 88].

Figure 2 shows several typical magnetoplasmonic nanostructures. From an optical mode point of view, magnetoplasmonic devices can be categorized as localized surface plasmon resonance (LSPR) modes (Figure 2(a)), which propagate SPR modes (Figure 2(b)), and hybrid resonance modes (Figure 2(c)). Nanostructures based on noble metal and ferromagnetic materials supporting LSPR have been widely studied both theoretically and experimentally [28, 29, 78], [79], [80]. Enhanced MO effects are observed owing to the electromagnetic field enhancement in the MO materials because of the LSPR resonance (pure MO contribution), and the decreased transmission/reflection of incident light (pure optical contribution) [23]. For example, the simultaneous enhancement of the MO activity and reduction of the optical absorption loss are demonstrated in a judiciously designed sandwich layer with Au, SiO_2_, and Co films by Banthi et al. [28] as shown in Figure 2(a). The propagating SPR (PSPR) modes have been studied to enhance the Faraday effect or MOKE [43, 81], [82], [83], [84], [85], [86]. As shown in Figure 2(b), Maccaferri et al. proposed 2D magnetoplasmonic crystals composed of Fe_20_Ni_80_ periodic nanoholes, which enhance the MO longitudinal Kerr effect by exciting the grating-coupled SPR mode [44]. Hybrid modes, wherein LSPR or PSPR modes are hybridized with other modes in the nanostructure, such as waveguide modes or lattice modes, have also attracted significant research interest [31, 36], [37], [38], [39, 87], [88], [89]. Narrower resonance peaks are observed because of the reduced scattering or absorption loss by exciting these hybrid modes. This leads to a stronger MO effect enhancement. For example, as shown in Figure 2(c), Kataja et al. studied the hybrid mode of the LSPR and lattice modes in periodic Ni nanodisks, and this yielded a Fano-type surface lattice resonance (SLR) mode [87]. The polar MOKE of the structure was governed by the SLR mode, and this was related to the polarization of the incident light parallel to periodicity. In another example shown in Figure 2(d), Chin et al. studied the enhancement of the Faraday effect in an Au grating on bismuth iron garnet (BIG) thin film nanostructures. They observed an 8.9 times improvement in the Faraday rotation (FR) together with a relatively high transmission of 36% at a wavelength of 963 nm by exciting the hybrid SPR and waveguide modes [31].

**Figure 3:**
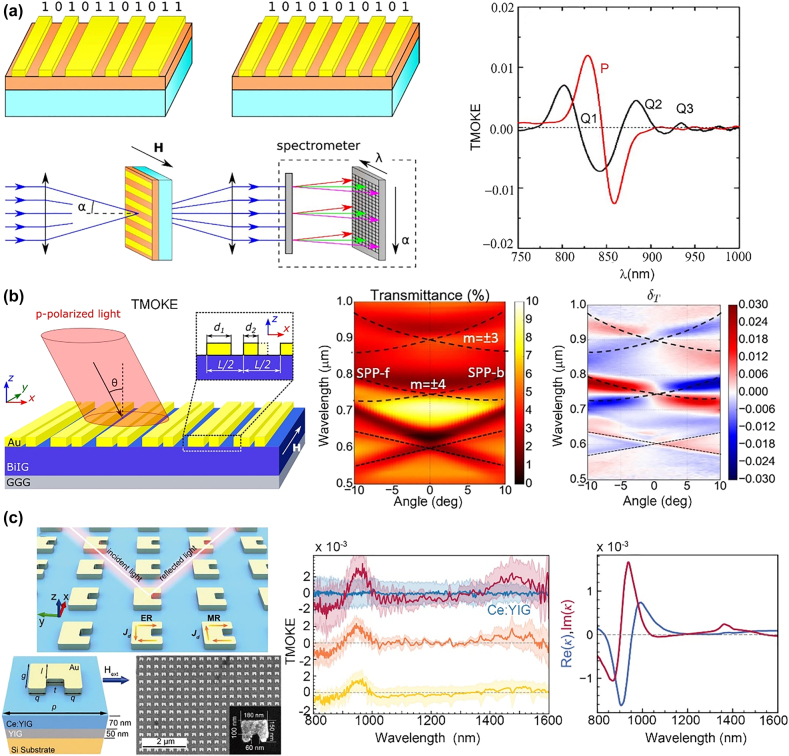
Recent developments of magnetoplasmonic devices with aperiodic/asymmetry structures. (a) Magnetoplasmonic quasicrystals with multiband enhancement of the MO response. The right figure presents TMOKE spectrum for periodic (red curve) and quasiperiodic structures (black curve). (b) Magnetoplasmonic crystals with broken spatial and time symmetry. The middle and right figures show the transmittance and TMOKE spectra as a function of the wavelengths and incident angles. A large TMOKE is observed under normal incidence at the SPR wavelength. (c) Magnetoplasmonic nanostructures with asymmetric structure (splitting ring resonator, SRR). The middle figure displays the s-polarized TMOKE under 45° (red), 50° (orange) and 55° (yellow) incidence. The right figure presents the equivalent off-diagonal elements of the permeability tensor of metamaterials at near-infrared wavelengths. The panels of this figure contain pictures adapted from References [92], [93], [94].

Novel magnetoplasmonic nanostructures and resonance modes have been recently reported. Although these modes can be categorized in the modes in Figure 2, unique MO effects are observed because of the aperiodic/asymmetric structure or mode profiles. The dark mode, wherein the radiative damping is strongly suppressed because of the antisymmetric field distributions, can further increase the MO effects. Dark modes are studied for magnetoplasmonic devices. Frolov et al. used dark surface lattice modes (SLMs) in Au/Ni/Au one-dimensional magnetoplasmonic crystals to enhance the transverse MOKE (TMOKE). The TMOKE of dark SLMs is almost three times higher than that of bright SLMs [90]. In the same year, Lopez-Ortega et al. used low-radiative multipolar dark modes in magnetoplasmonic nanocavities to achieve a large enhancement of the MO-induced modulation of light polarization, and this presented one order of the magnitude enhancement of field intensity compared to the bare magnetoplasmonic nanoantennas [91]. Aperiodical and asymmetric plasmonic nanostructures have been proposed in several studies. These structures can enable modes that differ from single dipole resonances or guided modes, which enables unique MO effects. In 2018, Kalish et al. proposed the concept of magnetoplasmonic quasicrystals, where the plasmonic gratings are no longer periodic, and this is similar to a quasicrystal, as shown in Figure 3(a). This device features a unique multiband MO response [92]. In 2020, Borovkova et al. investigated magnetoplasmonic structures with broken spatial symmetry and discovered notable MO modulations of the transmitted light at normal incidence with in-plane magnetization, this is forbidden in conventional symmetric nanostructures, as shown in Figure 3(b) [93]. Recently, Yang et al. proposed a nanostructure comprising Au split-ring resonators on a Ce:YIG film, as shown in Figure 3(c) [94]. The LSPR modes excite the electric field orthogonal to the incident light polarization direction, which leads to unique s-polarized TMOKE, which is not observed in natural MO materials at optical frequencies. Further, the authors retrieved the effective permittivity and permeability tensors of the metamaterial, which showed the bi-gyrotropic nature of the magnetoplasmonic nanostructure.

**Figure 4:**
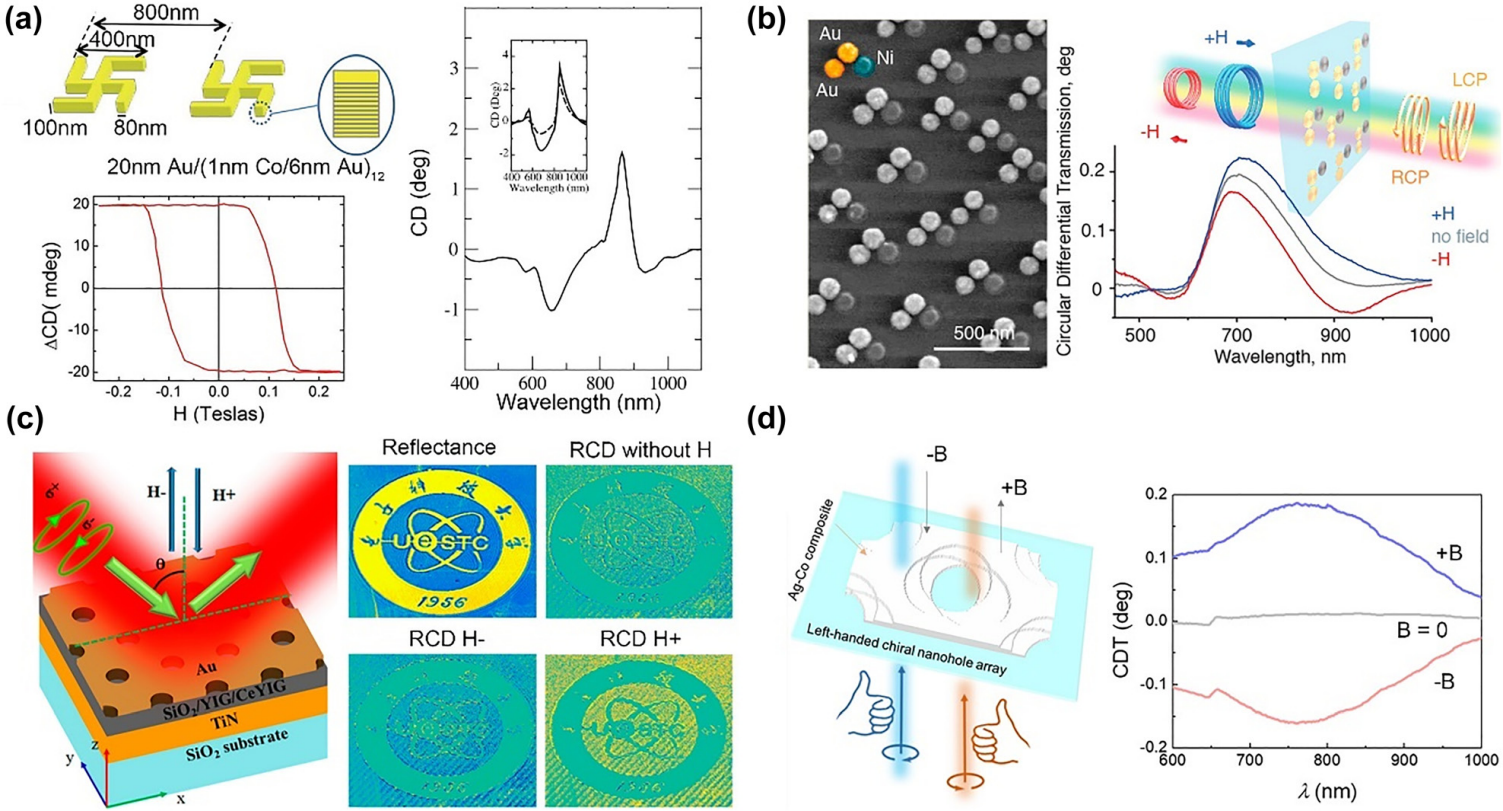
Chiral magnetoplasmonic devices. (a) Chiral magnetoplasmonic metasurface based on Au/Co multilayers shaped with gammadion crosses. A large CD (1.5 deg) presented in the gammadion crosses with magnetic tunable CD up to ±20 mdeg. (b) Chiral magnetoplasmonic metasurface based on Au–Au–Ni trimers. The circular differential transmission shows large variation under positive, negative, and zero applied magnetic fields. (c) Chiral magnetoplasmonic metasurface based on Au periodic nanohole/Ce:YIG/YIG/SiO_2_/TiN multilayers. Chiral imaging modulated by the magnetic field. (d) Chiral magnetoplasmonic metasurface based Ag/Co chiral nanohole arrays with in-plane symmetry breaking. A large magnetic field tunable circular differential transmission is demonstrated in this magnetoplasmonic chiral structure. The panels of this figure contain pictures adapted from References [99, 101], [102], [103].

An interesting research direction for chiral magnetoplasmonics has recently gained attention. A structure is called chiral when its mirror image cannot overlap with itself through translation and rotation operations; such chiral plasmonic nanostructures are interesting because they can enhance the circular dichroism (CD) signals of enantiomers, and it is important for chiral molecule sensing. Unlike natural chiral molecules with fixed chiroptical properties, the chirality of chiral plasmonic nanostructures can be modulated by an external stimulus such as temperature, strain, magnetic field, and light, which is useful for chiroptical sensing [95], [96], [97], [98], [99]. Chiral magnetoplasmonics perfectly fit this scenario and magnetic field tunable plasmonic nanostructures can achieve high modulation speeds and large modulation amplitudes. Driven by an applied magnetic field, they do not generate heat or local chemical reactions. In 2014, Armelles et al. proposed a chiral magnetoplasmonic structure of gammadion crosses comprising Au/Co multilayers. The author observed a large CD of up to 1.5° at an LSPR wavelength of 850 nm. Further, they demonstrated a modulation of the CD up to ±20 mdeg by an applied magnetic field of 2 kOe (Figure 4(a)) [99]. The same group proposed a nanostructure with chiral plasmonic oligomers and a separated Au/Co multilayer in the next year [100]. The mutual interaction strength between the MO and chiro-optical effects can be tuned by changing the thickness of the separating layer, this opens the door for the fabrication of magneto-chiral systems over a wide spectral range. In 2018, Zubritskaya et al. devised Au–Au–Ni trimer nanoantennas with magnetic and chiral properties. The structure showed the modulation amplitude of more than 100% for the magnetically tunable chiroptical response [101], as indicated in Figure 4(b). In 2020, Qin et al. proposed a chiral magnetoplasmonic metasurface based on MO oxide material (Ce_1_Y_2_Fe_5_O_12_) and Au periodic nanoholes [102], as indicated in Figure 4(c). The authors observed the continuous magnetic field modulation of the extrinsic chiroptical properties of the devices. The measured CD varied in a large amplitude range from −0.65° to +1.9°. Further, they demonstrated magnetic field tunable chiral images in a 2 mm × 2 mm large device fabricated by lithography and self-assembly. Jeong et al. proposed self-assembled chiral nanoparticles using magnetoplasmonic Ag@Fe_3_O_4_ core–shell nanoparticles in helical magnetic fields. The structure showed the dynamic switching of CD(±3°) by *h*B in millisecond time [54]. In 2021, Luong et al. proposed an Ag/Co composite chiral nanohole array with in-plane symmetry breaking [103], as shown in Figure 4(d). The maximum modulation of the CD by an applied magnetic field can reach 0.35°. Recently, Petrucci et al. proposed a magneto-chiroptical metasurfaces consist of spiral arranged one Ni and four Ag nanodisks [104]. Due to strong interaction between magnetic (Ni) and plasmonic (Ag) nanodisks, large non-reciprocal and reciprocal CD were demonstrated. A chiral structure shows broken spatial reversal symmetry (parity), whereas a magnetic material shows broken time reversal symmetry (time). Although they both manifest themselves by optical CD in the far field, only structural chirality leads to near-field optical chirality in plasmonic nanostructures [102]. This difference makes such systems interesting in physics, and they are also attractive for sensing applications that allow more degrees of freedom to control the near-field and far-field chiroptical properties independently.

## 4 All-dielectric MO nanostructures

### 4.1 All-dielectric MO subwavelength gratings

In contrast to magnetoplasmonic devices, MO nanophotonic structures comprising all-dielectric materials do not exhibit absorption issues caused by noble and ferromagnetic metals. Therefore, a considerably higher quality factor for the resonance modes can be achieved. This research direction has recently attracted significant interest.

**Figure 5:**
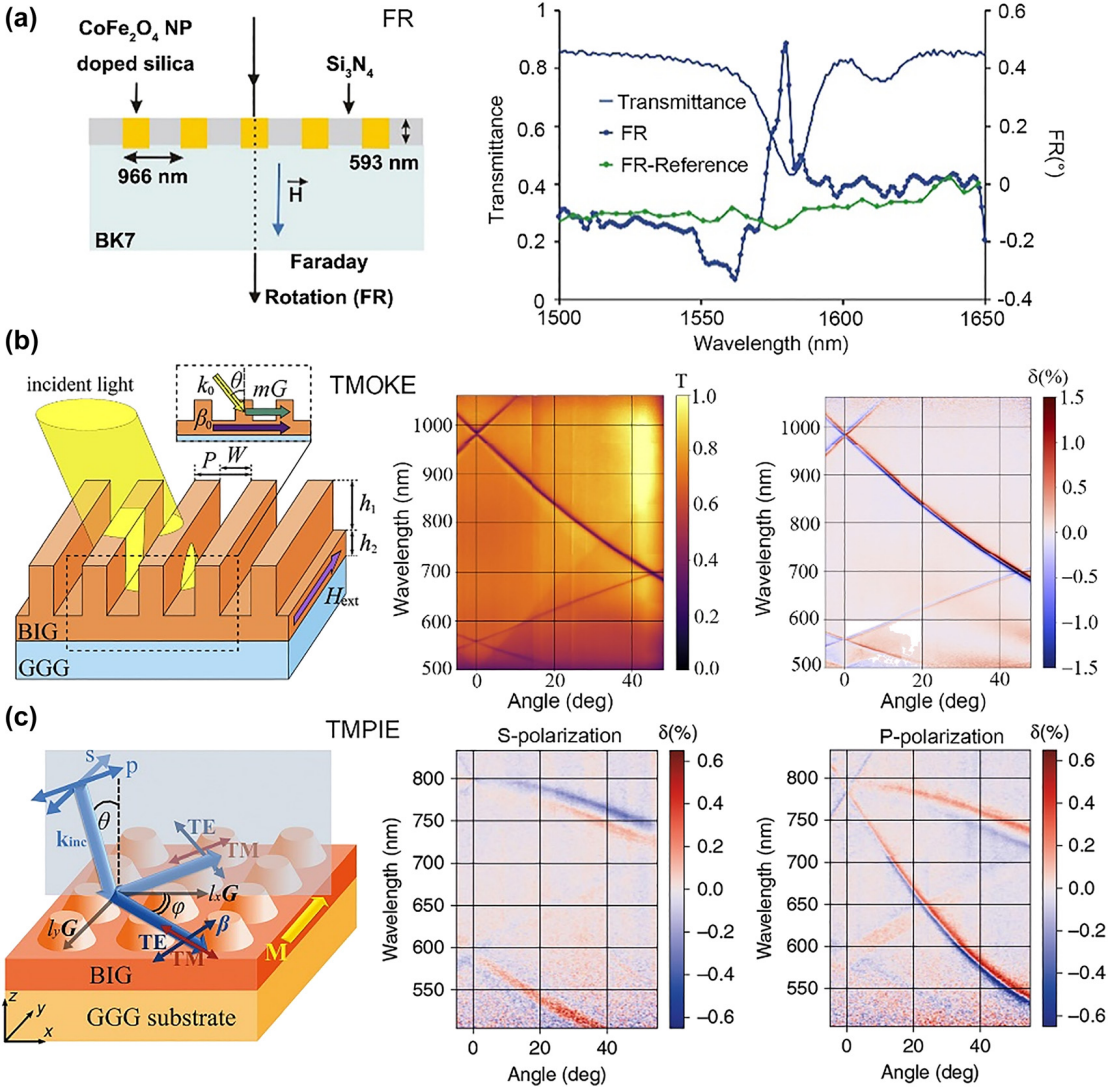
Waveguide modes in all-dielectric MO subwavelength gratings. (a) SiN/CoFe_2_O_4_ nanoparticle doped SiO_2_ grating and the enhanced Faraday effect. The right figure presents the transmission and FR spectra, showing large FR enhancement at the Mie resonance wavelength. (b) Structure, transmittance, and TMOKE in BIG 1D subwavelength grating structures on gadolinium gallium garnet (GGG) substrates. (c) Structure and TMPIE for both s and p polarized incidences of 2D BIG subwavelength gratings on GGG substrates. The panels of this figure contain pictures adapted from References [62, 105, 106].

All-dielectric MO subwavelength gratings supporting high-Q guided modes were first proposed theoretically [57, 105, 106]. In 2006, Bai et al. designed an all-dielectric MO grating based on a high-index Bi:YIG material, which achieves 90° Kerr rotation when exciting the grating-coupled guided-mode resonance [107]. Here, the author assumed lossless Bi:YIG materials. Therefore, the gigantic Kerr angles can only take place in calculations. In 2014, Marsymov et al. theoretically demonstrated a large enhancement of TMOKE in all-dielectric gratings composed of Bi:YIG/Si periodic nanostripes when the guided modes were coupled with Wood’s anomaly [59]. Due to the high index of Si, the gratings can achieve high reflectivity (96%) at the maximum of the TMOKE response wavelength. Subsequently, Gamet et al. proposed a similar all-dielectric grating in 2017, and this theoretically demonstrated the enhancement of MO effects in Faraday, PMOKE, LMOKE, and TMOKE configurations [108]. The MO material was a silica matrix doped with cobalt ferrite (CoFe_2_O_4_) nanoparticles, and the nonmagnetic dielectric was Si_3_N_4_. This group fabricated the device in 2020 and experimentally observed a fivefold enhancement of the FR for the TE/TM phase-matching scenario, as shown in Figure 5(a) [105]. Although a 0.49° FR angle was achieved, the low level of transmittance was the main drawback. In 2020, Bsawmall et al. fabricated a dielectric grating (photoresist) on top of an MO layer (tetraethyl orthosilicate doped with cobalt ferrite nanoparticles) and experimentally demonstrated a significant enhancement of the LMOKE up to 1.1° at a wavelength of 1506 nm [61]. In the same year, Voronov et al. experimentally demonstrated three orders of magnitude higher TMOKE in all-dielectric BIG subwavelength gratings compared to the unpatterned BIG film, partly resulted from the particularly small MO effect of the unpatterned BIG film (Figure 5(b)) [106]. The structure can support the propagation of TM-guided modes with a high quality factor *Q* = *λ*/∆*λ* = 138, which is an order of magnitude higher than plasmonic resonant structures. A high transmittance of *T* = 60% and a large TMOKE reaching 1% were simultaneously demonstrated. Later, authors proposed a two-dimensional (2D) all-dielectric BIG MO grating and observed MO light intensity modulation in the transmission for both s-and p-polarized incident light when exciting the TE-and TM-guided modes, i.e., the transverse magnetophotonic intensity effect (TMPIE), which is absent in smooth magnetic thin films, as shown in Figure 5(c) [62]. For the TE mode, the measured intensity modulation by magnetization can reach *δ* = 0.3%, together with a high transmittance of *T* = 55%. Research on hybrid-mode-enhanced MO effects starts to emerge in all-dielectric nanostructures. Recently, hybrid modes between localized (Fabry–Perot like) and lattice (guided-like) modes have been observed in a 2D iron-garnet nanocylinder array by Belotelov et al. The excitation of this mode leads to a three times enhancement of the FR and an order of magnitude enhancement of the TMOKE compared to the magnetic film with equal thickness [109].

**Figure 6:**
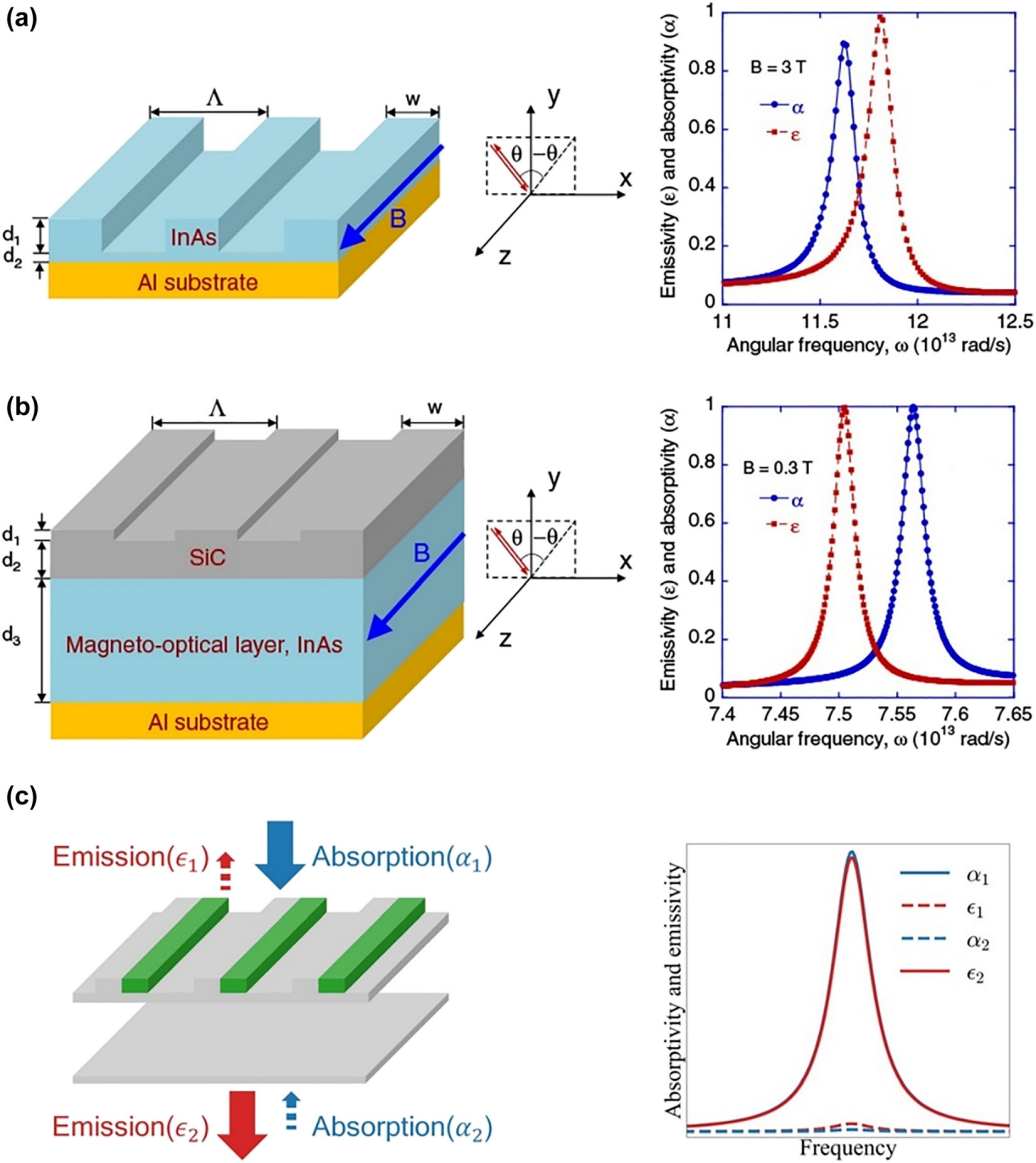
Violation of Kirchhoff’s law of thermal radiation in all-dielectric MO subwavelength gratings. (a) Structure, absorption, and emission spectra of InAs gratings on an Al substrate under 3 T applied magnetic field. The emission and absorption spectra present different peak positions under the applied magnetic field. (b) Structure, absorption, and emission spectrum of SiC grating on InAs film on an Al substrate under 0.3 T applied magnetic field. The emission and absorption peaks separate under the applied magnetic field. (c) All-dielectric MO grating for violation of the Kirchhoff’s law of thermal radiation in the transmission mode. Large transmission difference of absorption and emission is observed under an applied magnetic field. The panels of this figure contain pictures adapted from References [63, 64, 110].

Extending the concept of all-dielectric MO gratings to the mid-infrared wavelength range leads to another fascinating research direction for nonreciprocal thermal emission. An MO thermal emitter can violate Kirchhoff’s law of thermal radiation; however, subwavelength grating structures can boost this effect because of the enhanced MO effect. Such systems are attractive for energy harvesting because they only absorb energy from but without re-emission from the thermal radiation source. In 2014, Zhu et al. theoretically proposed a structure composed of InAs gratings on an Al substrate, and this can result in near-complete violation (i.e., difference between emissivity and absorptivity at a given frequency and direction can reach near unity) of Kirchhoff’s law under an applied magnetic field of 3 T, as indicated in Figure 6(a) [110]. In order to reduce the applied magnetic field, Zhao et al. theoretically proposed an all-dielectric MO grating structure with SiC periodic gratings on InAs films, as shown in Figure 6(b). A near-complete violation of Kirchhoff’s law can be achieved under only 0.3 T applied magnetic field by exciting the grating-coupled guided modes in this device [63]. However, in their calculation, the electron mobility of the InAs material reaches ∼3 × 10^5^ cm^2^/(Vs), which is difficult to fabricate in experiments. Recently, Park et al. proposed a semitransparent 1D grating structure to violate Kirchhoff’s law in the transmission. The structure can achieve a significant contrast between absorptivity and emissivity by exciting the guided-mode resonance, as shown in Figure 6(c) [64]. Magnetic Weyl semimetals have been proposed in grating structures to solve the large applied magnetic field problem [111, 112]. Materials such as EuCd_2_As_2_ are proposed as promising candidates for realizing a nonreciprocal thermal emitter with no applied magnetic field [113]. An experimental demonstration of these proposals is an exciting research direction for future work.

### 4.2 Mie resonance modes in all-dielectric MO resonators

**Figure 7:**
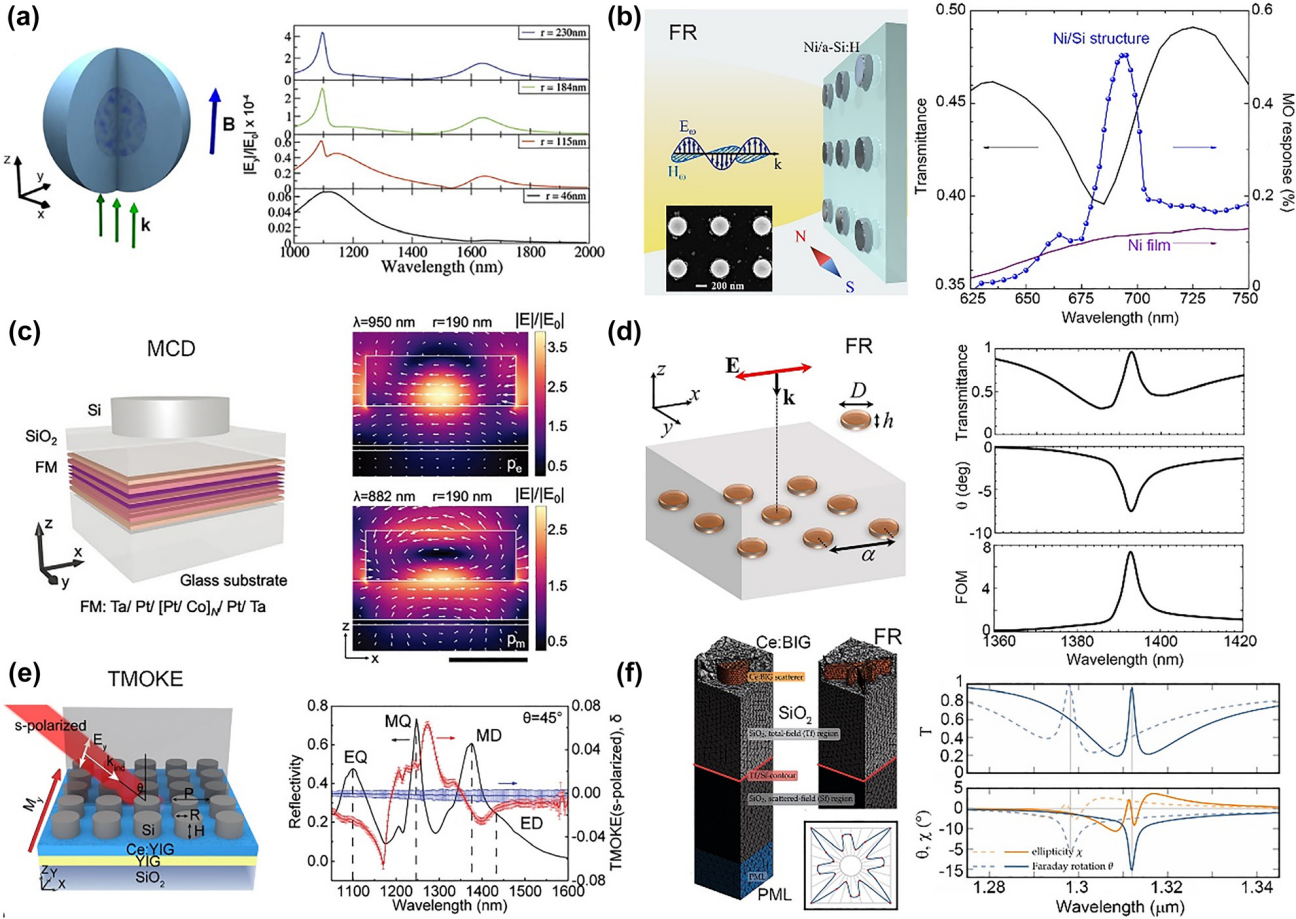
All-dielectric MO resonators with Mie resonances. (a) All-dielectric MO structure composed of Si spherical with a dielectric MO material core. The right figure presents the polarization rotation for different magnetic core radii: 230 nm, 184 nm, 115 nm and 46 nm. (b) All-dielectric MO structure based on the array of α-Si nanodisks covered by a 5 nm-thick nickel film. The right figure presents the experimental transmission (black, solid) and MO response (blue, circles). (c) All-dielectric MO structure based on *α*-Si nanodisks on ferromagnetic metallic multilayers. The right figure presents the electric field magnitude and direction maps of disk cross-sections at electric (top) and magnetic (bottom) resonances for disk radius of 190 nm. (d) All-dielectric MO structure with BIG periodic nanodisks, which supports the EIT modes. The right figure presents the transmission (*T*), FR (*θ*) and FOM (√*T*|*θ*|) spectra when EIT is excited. (e) All-dielectric MO nanoresonators composed of Si nanodisks on Ce:YIG thin films. S-polarized TMOKE is observed, which is absent in a bare Ce:YIG film. The right figure presents the reflectivity (black curve), s-TMOKE spectrum (red curve) of the metasurface, and the s-TMOKE spectrum of the bare Ce:YIG film (blue curve). (f) All-dielectric MO structure based on Ce:BIG scatterers optimized by Bayesian algorithm. The right figure presents the spectra for transmittance, FR and ellipticity. The panels of this figure contain pictures adapted from References [65, 66, 114, 117], [118], [119].

Mie resonance modes can occur in both metallic and dielectric nanoparticles when their sizes are comparable to the incident light wavelength. Recent research interest has been focused on Mie resonance modes in dielectric nanoresonators because of their lower loss and more complex modes, which includes magnetic resonance modes compared to metallic nanoparticles. All-dielectric MO resonators supporting Mie resonance modes have been studied recently. In 2016, de Sousa et al. first calculated the MO activity in high-index dielectric silicon nanoparticles with an MO core, as indicated in Figure 7(a) [65]. This work demonstrates that the MO response is fully governed by magnetic dipolar and quadrupolar resonances. The electric resonance modes showed very little effect on the MO activity because of the lower field intensity in the MO core material, and this was different from the plasmonic resonances. The first experiment was conducted by Barsukova et al. who utilized a hybrid nanostructure comprising periodically arranged amorphous Si (*α*-Si) nanodisks and nickel thin magnetic film (Figure 7(b)) [114]. The MO response of the hybrid nanostructure can achieve an obvious enhancement near the magnetic dipole resonance of nanodisks compared with the bare 5 nm-thick nickel films. The same structure was numerically simulated by Musorin et al., who demonstrated a significant enhancement of the MO response when the electric and magnetic dipolar resonances were overlapped spectrally [115]. In 2019, a similar device composed of hydrogenated *α*-Si nanodisks covered with a 5 nm-thick nickel film was experimentally demonstrated to achieve the multifold enhancement of the Faraday effect in the spectral vicinity of the magnetic dipole resonance [116]. A characteristic value of the FR angle observed in this structure is *θ* = 0.8°. Limited by the large optical absorption of the nickel films, the transmittance of the all-dielectric resonator was low. Although these all-dielectric nanostructures are shown to boost MO effects with linearly polarized incident light, Abendroth et al. demonstrated enhanced magnetic CD by Mie resonance structures comprising amorphous Si nanodisks on Ta 3/Pt 3/[Pt 0.7/Co 0.6]_N_/Pt 3.7/Ta 3 (in nm) multilayer films (Figure 7(c)). These films demonstrate magnetic field-induced dissymmetry over ±2% for circularly polarized incident light [117]. In contrast to the unpatterned background that exhibits a modest wavelength-dependent dissymmetry, the patterned structure showed a six-fold enhancement of local electric field rotation within the proximal ferromagnetic films for circularly polarized light excitation, which highlights the influence of Mie resonances in the structure on the MO response. To improve the transmittance, Christofi et al. utilized the BIG nanodisk arrays to numerically demonstrated a large FR enhancement and high transmittance attributed to electromagnetically induced transparency (EIT), as shown in Figure 7(d) [118]. They theoretically demonstrated the enhancement of both the FR and MO FOM by carefully engineering the electric and magnetic dipolar resonance modes to overlap. Recently, Xia et al. theoretically proposed and experimentally fabricated a structure composed of periodically arranged Si nanodisks MO oxide Ce:YIG films (Figure 7(e)) [66]. They observed anomalous MO effects, which includes giant s-polarized TMOKE (*δ* = 6.4%) and two orders of magnitude higher LMOKE under near-normal incidence conditions compared to a bare Ce:YIG film. These anomalous MO effects are attributed to the unique circular displacement current when exciting the magnetic Mie resonance modes, this locally changes the electric field direction in the structure. In 2021, Kiel. et al. optimized the MO response of an all-dielectric Huygens metasurface comprising MO nanodisks of cerium-doped BIG using a Bayesian optimization algorithm (Figure 7(f)) [119]. The optimal structure showed 100% transmittance and a 15° FR angle, and this demonstrated the promising potential of all-dielectric MO Mie resonators for free-space nonreciprocal photonic device applications.

**Figure 8:**
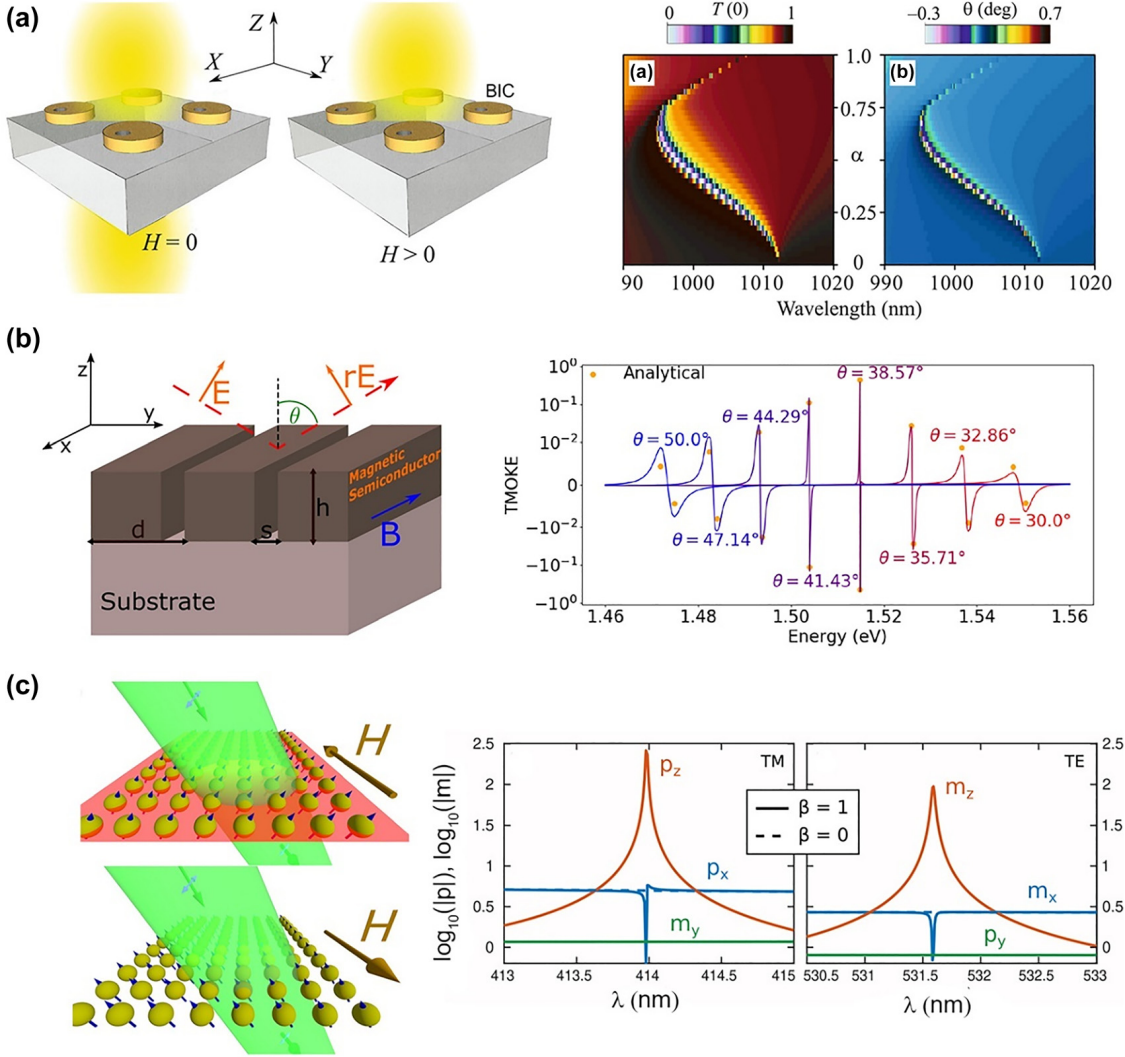
All-dielectric MO resonators with BIC resonance modes. (a) Structure, transmittance, and FR in all-dielectric BIG nanodisks with air holes displaced away from the disk center. The right figure presents the transmission and FR spectra as function of the asymmetry factor *α*. (b) Structure and TMOKE in a magnetic semiconductor Cd_*x*_Mn_1−*x*_Te grating that supports BIC modes. The right figure presents the TMOKE spectra for different incident angles. (c) Tuning of BIC using applied magnetic field in all-dielectric MO structure. The middle figure presents the out-of-plane electric dipole *p*_*z*_ and in-plane contributions *p*_*x*_, *m*_*y*_ for TM polarization with (*β* = 1, solid curves), and without (*β* = 0, dash curves) MO activity. The right figure presents the out-of-plane magnetic dipole *m*_*z*_ and in-plane contributions *m*_*x*_, *p*_*y*_ for TE polarization. The panels of this figure contain pictures adapted from References [121], [122], [123].

A large radiation loss exists although all-dielectric resonators exhibit low optical absorption loss. The bound state in continuum (BIC) mode has been proposed recently to reduce radiation loss. The BIC refers to a singular state that remains perfectly confined despite lying in a continuous spectrum of radiation, and it has an infinite lifetime with an ultrahigh *Q*-factor [120]. The interaction between the BIC mode and MO materials in all-dielectric resonators can lead to a significant enhancement of the MO effects. In 2020, Chernyak et al. theoretically analyzed the enhancement mechanisms of the MO effects in an all-dielectric nanostructure caused by the BIC resonance [121]. As shown in Figure 8(a), the device comprises Bi:YIG nanodisks with air holes displaced away from the disk center. The FR can reach 0.7° because of the high-*Q* (*Q* ∼ 3100) resonance of the BIC. In the same year, Zakharov et al. theoretically proposed an MO grating structure comprising a magnetic semiconductor Cd_*x*_Mn_1−*x*_Te that can support the BIC resonance by changing the incident angles (Figure 8(b)) [122]. A significant TMOKE strength reaching unity was demonstrated when BIC resonance was excited. In 2021, Abujetas et al. analytically and numerically investigated the tuning of the quasi-BIC mode in MO all-dielectric metasurfaces (Figure 8(c)) [123]. According to the formulation of coupled electric/magnetic dipole resonances proposed by the authors, the quasi-BIC mode in this system was attributed to the interference of the out-of-plane electric/magnetic dipole resonances with the MO-induced in-plane magnetic/electric dipole. Therefore, the MO activity can be utilized to tune and switch the quasi-BIC mode. Although the BIC mode presented large MO effects and novel MO phenomena, experimental work still faces challenges due to the high precision requirement of the nanofabrication and the difficulty of the etching process of the MO oxides.

## 5 Applications

### 5.1 Biosensing and chemical sensing

**Figure 9:**
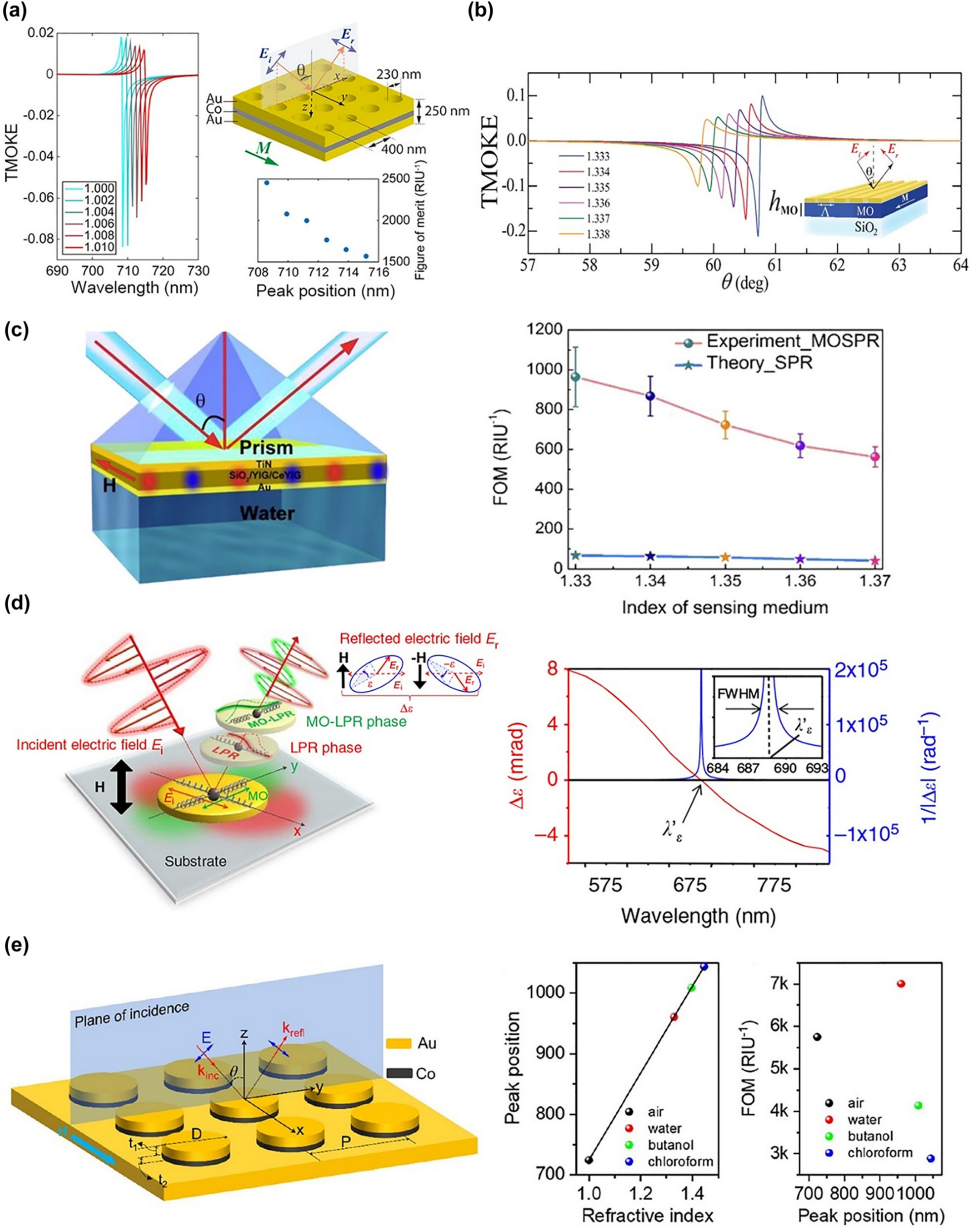
Applications of magneto-nanophotonic devices for bio/chemical sensing. (a) MOSPR biosensors based on the perforated nanoholes. The FOM reaches 2500 RIU^−1^ using TMOKE as signal for biosensing. (b) MOSPR biosensors using hybrid Au grating and ferromagnetic metal film. The angular spectra of TMOKE present high sensitivity to the medium index change. (c) MOSPR biosensors based on low loss magnetic oxides. The right figure presents the measured FOM as a function of the index of sensing medium for MOSPR biosensor. (d) MOSPR biosensors based on random Ni nanodisks. The right figure displays the ellipticity variation (Δ*ε*, red line) and 1/Δ*ε* (blue line) as a function of the wavelength. The close-up view of the 1/Δ*ε* spectrum in the inset shows a narrow full width at half-maximum (FWHM) (∼1.7 nm). (e) MOSPR biosensors based on periodic arranged Au/Co nanodisks. The middle and right figures display the resonant peak position as a function of the index of the sensing medium, and FOM for different sensing mediums. The panels of this figure contain pictures adapted from References [53, 85, 86, 133, 136].

The SPR is widely used for label-free biosensing and chemical sensing [124, 125] because of the strong localization of the electric field at the interface of the metal sensitive to the surrounding medium variation. However, the large optical loss of the metals leads to a wide resonance peak of the SPR, which limits the sensitivity and limit of detection (LOD) of the sensor. In comparison to SPR, MO surface plasmon resonance (MOSPR) combines the SPR and MO activity of magnetic materials, and this can enhance the TMOKE at the resonance wavelength. In this case, the MOSPR can use the narrowband MO response as the signal of sensing instead of the broad-band transmission or reflection signals. Therefore, MOSPR shows a higher FOM and lower LOD than the SPR biosensors. The first MOSPR sensor was proposed by Sepúlveda et al. who demonstrated a threefold improvement in the LOD compared to the standard SPR sensors in Co/Au heterostructures [126]. Subsequently, MOSPR with different noble metals and ferromagnetic metals for biosensing has been widely studied, e.g., Au/Co/Au [86, 127, 128] and Au/Fe/Au [129], [130], [131] trilayers. In these works, a careful theoretical analysis of the optimum multilayers was carried out to achieve maximum device sensitivity and FOM, with a focus on balancing the trade-off between MO activity and optical absorption. In 2016, Ignatyeva et al. proposed a magnetoplasmonic sensor combining a one-dimensional photonic crystal and a ferromagnetic cobalt layer [132]. The heterostructure can excite an ultralong propagating MOSPR mode with a propagation length of up to 106 μm, and this shows an extremely sharp SPP (angular width 0.06°) and MOSPR resonance (angular width 0.02°). A high-index prism is required to excite the SPR mode, which limits the devices for on-chip integration, because of the phase mismatch between free-space light and SPR. Exciting SPR with gratings has been widely studied to reduce the size of MOSPR sensors. Caballero et al. carried out a theoretical study on Au/Co/Au multilayers perforated with a periodic array of nanoholes. The FOM of the proposed MOSPR device is two orders of magnitude larger than that of a plasmonic sensor, as shown in Figure 9(a) [86]. Diaz-Valencia et al. proposed a one-dimensional magnetoplasmonic crystal comprising a periodic metal grating grown on an MO metallic substrate with a sensitivity of 190° RIU^−1^ and an FOM of the order of 10^3^ (Figure 9(b)) [85]. The large optical loss of ferromagnetic metals limits the FOM and LOD. To solve this problem, magnetic oxides such as cerium/bismuth-doped yttrium iron garnet (Ce:YIG/Bi:YIG) have been utilized in combination with noble metals because of the low loss and strong MO effects of these materials [11, 14]. Qin et al. proposed a metal–insulator–metal magnetoplasmonic sensor based on Ce:YIG magnetic films [133]. The sensor showed an ultrahigh FOM (964 ± 150 RIU^−1^) and low LODs (4.13 × 10^−6^) experimentally, which is 17.8× higher and 16× lower than that of a standard Au SPR sensor, respectively, by carefully designing the structure to excite a hybrid mode between the SPR mode at the Au/air interface and the MO waveguide mode in the Ce:YIG layer (Figure 9(c)).

Despite the high FOM of the MOSPR sensors, it remains difficult to achieve single-molecule sensitivity at the nanoscale. The LSPR modes excited by subwavelength structures have attracted considerable interest because of their higher surface sensitivity and point-of-care sensing capability. In 2011, Bonanni et al. proposed a magnetoplasmonic device that supports LSPR modes based on Ni nanodisks [27]. The inverse of the Kerr rotation was used as the detection signal, which presents a virtually unlimited value for the FOM (defined as the resonance shift divided by the FWHM of the resonance peak). In 2014, Maccaferri et al. experimentally demonstrated two orders of magnitude enhancement of the sensitivity compared to state-of-the-art magnetoplasmonic sensors by exploiting the control of the phase of light in Ni magnetoplasmonic nanoantennas. They reached a sensitivity of ∼0.8 ag per nanoantenna for polyamide-6.6 sensing, as shown in Figure 9(d) [53]. This phase-based sensing method was applied by other researchers to enhance the FOM of magnetoplasmonic nanogratings [134] and nanodisks [135]. Pourjamal et al. demonstrated an order of magnitude enhancement of the FOM in Ni/SiO_2_/Au dimer arrays compared to randomly distributed dimers, which results from the hybridization between the LSPR mode and lattice mode, to further increase the surface sensitivity [79]. More complex hybridized modes with propagating SPP, LSPR, and lattice modes were proposed by Li et al. in magnetoplasmonic sensors, and it showed a narrow Fano-like TMOKE spectrum with only sub-nanometer FWHM, as shown in Figure 9(e) [136].

### 5.2 Magnetic field sensing

**Figure 10:**
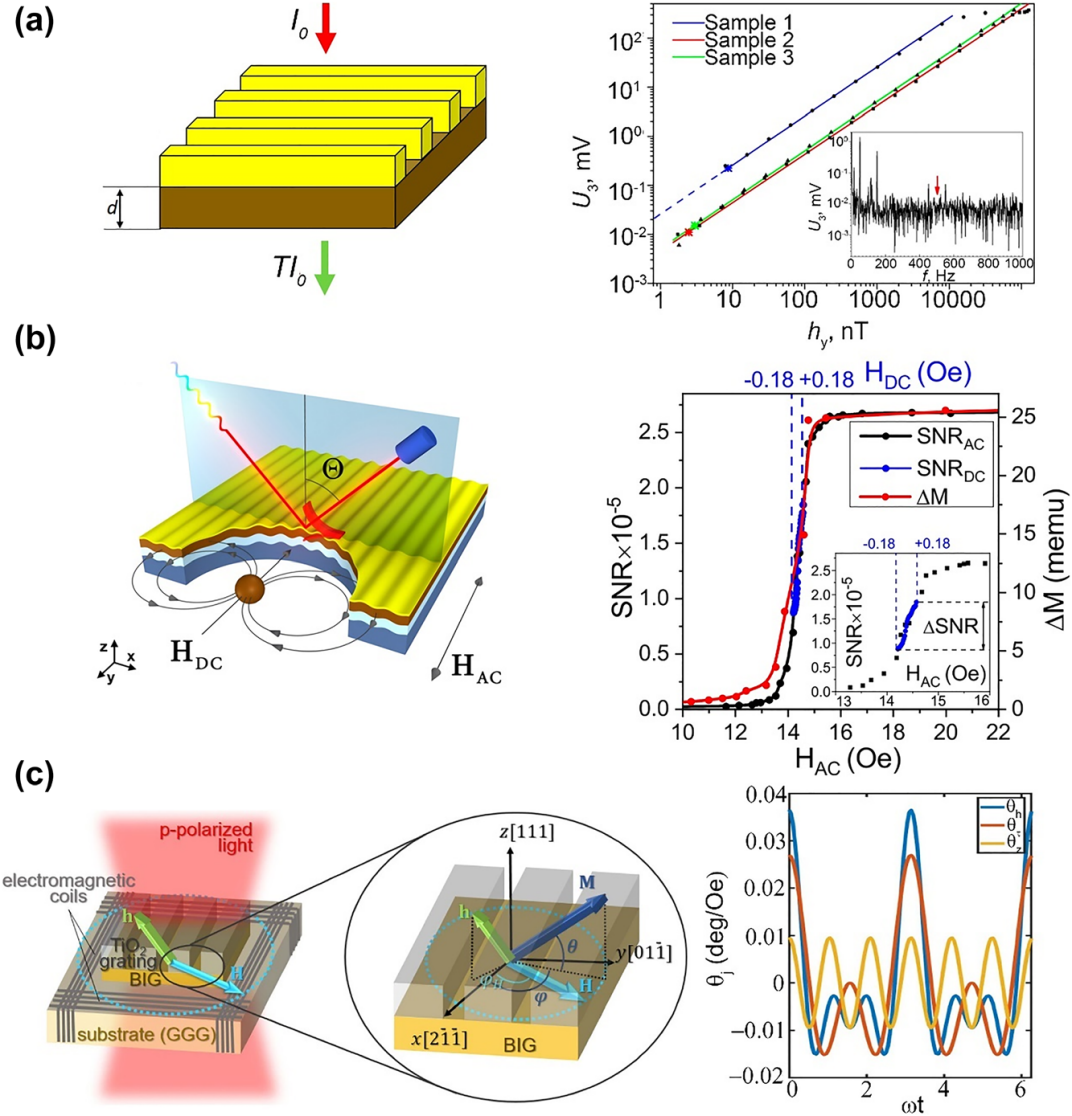
Applications of magneto-nanophotonic devices for magnetic field sensing. (a) A magnetometer based on the LMPIE in Au grating on bismuth doped yttrium iron garnet thin films. The right figure presents the third-harmonic amplitude *U*_3_ of the photodetector signal as a function of the applied magnetic field along the slits. The magnetic field oscillating at 515 Hz (indicated by a red arrow on the noise characteristic in the inset). (b) A magnetometer for direct current using an Ag grating on ferromagnetic metal Fe magnetoplasmonic device. The right figure presents the magnetic field dependence of the signal-to-noise ratio (SNR) and the relative changes of iron layer magnetic moment (Δ*M*). Blue dashed curves show the DC magnetic field range. (c) A magnetometer for vectoral field sensing based on all-dielectric TiO_2_ gratings on a BIG film. The right figure presents temporal dependence of the out-of-plane angle *θ* of the magnetization. The panels of this figure contain pictures adapted from References [47, 137, 138].

It is natural to use magneto-nanophotonic devices for magnetic field sensing because the optical properties of such devices are sensitive to the applied magnetic field. Knyazev et al. proposed a room-temperature magnetoplasmonic magnetic field sensor for this application [47]. The magnetometer operates on the principle of the LMPIE, which was first demonstrated by Belotelov et al. in 2013 [46]. A magnetic field is sensed by the variation of transmitted or reflected light intensity caused by magnetizing the film perpendicular to the grating slits. A schematic of the magnetometer is shown in Figure 10(a), it comprises an iron garnet film and a periodic gold grating. The sensitivity of the magnetometer reaches 2 nT/Hz^1/2^, which is limited by shot noise. Further, the sensitivity can reach the fT/Hz^1/2^ level if the shot noise is reduced. In 2020, Belyaev et al. proposed a magnetic field sensor to sense direct currents (DC) based on a magnetoplasmonic crystal [137]. The sensor measures the DC magnetic field component parallel to the AC magnetic field, which reaches a sensitivity level of 300 pT (Figure 10(b)). Ignatyeva et al. designed a vector MO magnetometer, as shown in Figure 10(c) [138]. The sensor comprises highly anisotropic iron garnet films and a one-dimensional TiO_2_ grating. The magnetometer was expected to show a high sensitivity of up to 100 pT/Hz^1/2^.

### 5.3 Magnetic field controlled active and nonreciprocal metasurfaces

**Figure 11:**
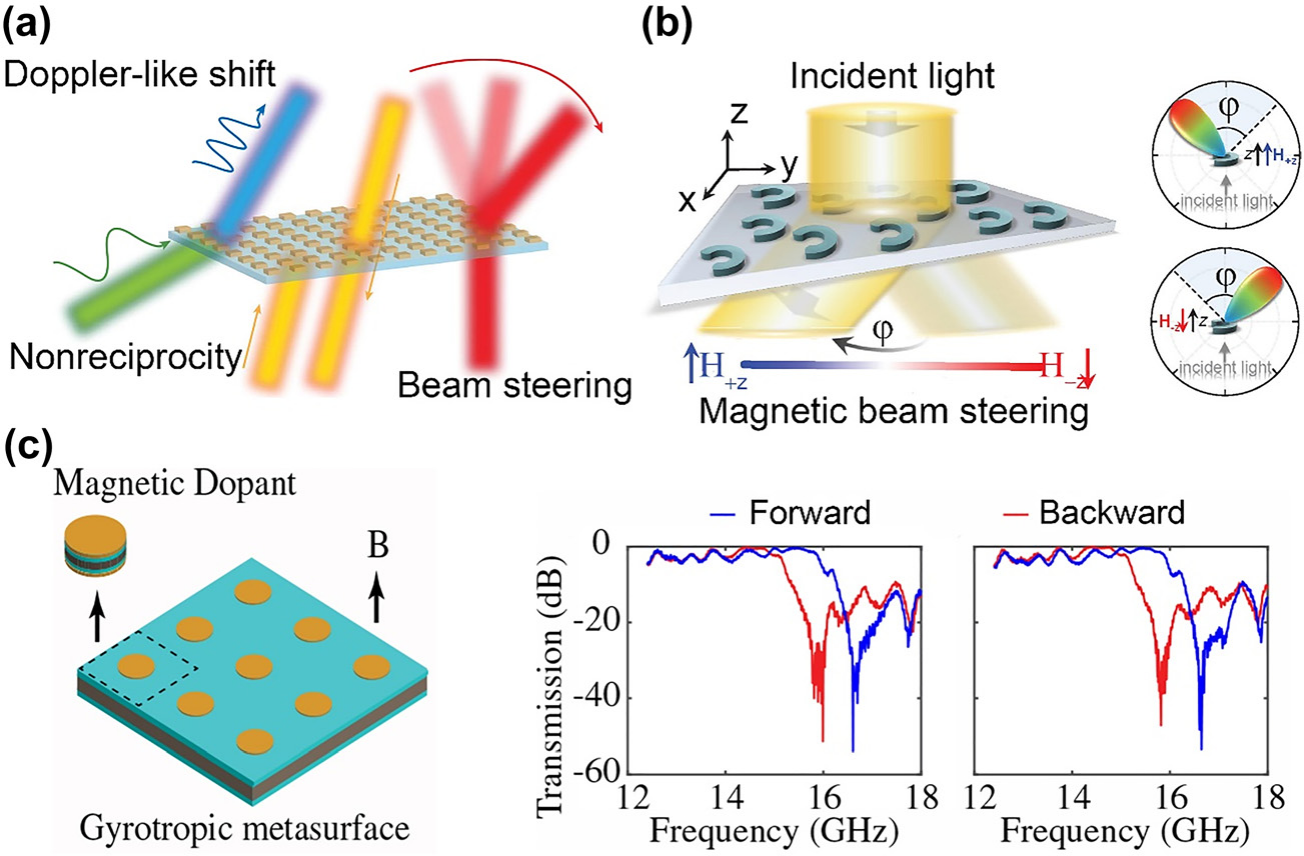
Magnetic field controlled active and nonreciprocal metasurfaces. (a) Schematic of spatiotemporal metasurfaces for Doppler-like shift, nonreciprocity, and beam steering. (b) An active metasurface based on BIG split-ring resonators for optical beam steering. (c) A gyrotropic nonreciprocal metasurface comprising YIG disks and its transmission spectrum in the microwave frequency. The panels of this figure contain pictures adapted from References [55, 139, 144].

Magnetic field-controlled active and nonreciprocal metasurfaces are important research directions for magneto-nanophotonic devices. Optical metasurfaces can be realized by designing plasmonic or dielectric nanostructures on a planar surface such that the local amplitude and phase of the reflected or transmitted light is sculptured, which can help obtain arbitrary far-field patterns. Magnetoplasmonic and all-dielectric MO nanostructures allow magnetic field-induced modulation of the amplitude and phase of each nanostructure, which adds the “time” degree of freedom. This concept of spatiotemporal active metasurfaces was first proposed in 2019 by Shaltout et al., this led to fascinating two-dimensional optical devices for wavelength conversion emulating a Doppler shift, nonreciprocal transmission, and active steering of the optical beams, as indicated in Figure 11(a) [55]. Magneto-nanophotonic metasurfaces fit perfectly in some of these applications. In 2020, Ihar et al. proposed an MO metasurface based on split-ring resonators made of BIG. These surfaces could realize optical beam steering using an applied magnetic field, as shown in Figure 11(b) [139].

A unique property of MO metasurfaces is nonreciprocity. In magneto-nanophotonic structures, nonreciprocity can be enhanced by the excitation of the resonance modes, which can allow the nonreciprocal transmission/reflection of electromagnetic waves in a subwavelength MO metasurface. Such nonreciprocal metasurfaces are proposed based on the optical nonlinearity and spatiotemporal modulation [140], [141], [142], [143]. Interestingly, there have been a limited number of studies on MO nonreciprocal metasurfaces. Liu et al. recently demonstrated nonreciprocal chiral transmission in gyrotropic disks with low magnetic fields in the microwave frequency range via experiments, as shown in Figure 11(c) [144]. At a resonant frequency of 15.68 GHz, the isolation ratio of the metasurface exceeds 40 dB for circular polarized incidence. Although in its infancy, magneto-nanophotonic metasurfaces demonstrate promising potential for the ultrafast, efficient, and nonreciprocal control of light propagation by magnetization. We expect the emergence of such devices in the future.

## 6 Conclusions and perspectives

We reviewed the recent developments and applications of nanophotonic devices based on MO materials. To this end, we summarized the recent progress of different magnetoplasmonic devices and all-dielectric MO nanophotonic devices based on the different modes of MO nanophotonic structures. Further, we discussed the recent research on the application of such nanostructures for bio/chemical sensing, magnetic field sensing, and active/nonreciprocal metasurfaces.

In future studies, several research directions should be considered.

1) All-dielectric MO nanostructures show promising potential for efficient light and magnetization interactions. MO effects induced by different resonant modes in all-dielectric nanostructures, such as anapole modes [145, 146] and supercavity modes [147, 148], require further theoretical and experimental investigations.
2) New phenomena and new applications that extend the MO nanophotonic concept to other frequencies, such as violation of Kirchhoff’s thermal radiation law in the mid-infrared, are expected to continue developing in the coming years.
3) The MO metasurfaces with nonreciprocal phase gradients are lacking, and they may allow arbitrary and nonreciprocal manipulation of the wavefront of the incident electromagnetic wave.
4) The magneto-nanophotonic structure may contribute to the topological and quantum photonics. For example, MO materials may play a crucial role in realizing nontrivial topology edge states in MO photonic crystals. MO nanophotonic devices may also realize single-photon nonreciprocity without hampering quantum coherence [149].

We believe that the rich physics between light and magnetization interaction at the nanoscale will make the above research directions an exciting field to explore in the years to come.
